# Clinicopathologic Correlation of Oral Lichen Planus and Oral Lichenoid Lesions: A Preliminary Study

**DOI:** 10.1155/2014/746874

**Published:** 2014-10-29

**Authors:** Marinka Mravak-Stipetić, Božana Lončar-Brzak, Iva Bakale-Hodak, Ivan Sabol, Sven Seiwerth, Martina Majstorović, Magdalena Grce

**Affiliations:** ^1^Department of Oral Medicine, Department of Oral Medicine, School of Dental Medicine, University of Zagreb, 10000 Zagreb, Croatia; ^2^Division of Molecular Medicine, Rudjer Boskovic Institute, 10000 Zagreb, Croatia; ^3^Department of Pediatric Dentistry, School of Dental Medicine, University of Zagreb, 10000 Zagreb, Croatia; ^4^School of Medicine, Institute of Pathology, University of Zagreb, 10000 Zagreb, Croatia

## Abstract

Oral lichen planus (OLP) and oral lichenoid lesions (OLL) are clinically and histologically similar lesions but their treatment planning and prognosis are different. The review of the literature indicates numerous criteria to distinguish these two lesions; however there is a lot of inconsistency. Thus, the aim of this study was to determine the correlation of histopathology and clinical OLP and OLL diagnosis and to clarify which histopathologic criteria could best distinguish these two diagnoses. A retrospective study showed that clinically diagnosed 92 OLPs and 14 OLLs have been confirmed histopathologically in 52.2% and 42.9% of cases, respectively. In addition, histopathology showed statistically significant more eosinophils (*P* < 0.0005), plasma cells (*P* < 0.0005), and granulocytes (*P* < 0.05) in OLL than OLP. To establish histopathological diagnosis of OLP and OLL it should be mandatory to define the type of cells in mononuclear infiltrate, which can be associated more accurately with clinical feature and patient history. Therefore, currently accepted diagnostic criteria for OLP and OLL should be modified and validated on a larger number of patients taking into account particular distinguishing histopathological features.

## 1. Introduction

Oral lichen planus (OLP) is a chronic immunological mucocutaneous disorder of unknown etiology with prevalence in general population ranging from 0.2 to 2% [1, 2], whereas the prevalence of OLP found in our population was 4.3% [3]. Due to low [2, 4, 5] and rather questionable [6–8] risk for malignant transformation, OLP is classified as potentially malignant disorder [9]. Indeed, OLP has distinctive clinical features within the oral cavity with several characteristic morphological types and symmetrical distribution in oral mucosa [5, 6, 8]. These features facilitate clinical diagnosis. However, due to its premalignant potential, nonreticular types, unilateral presentation, or lesions present at the cancer-risk oral sites it requires histopathological confirmation as well.

Histopathological criteria for OLP, which are currently accepted in clinical practice, are those given by WHO [10]. These criteria do not distinguish between the OLP and oral lichenoid lesions (OLL); therefore, several reports have suggested their modifications [8, 11, 12]. Van der Meij and van der Waal [11] proposed modified WHO criteria according to which OLP could be diagnosed only in cases when both clinical and histopathological criteria are fulfilled, while in other cases, the disorder should be considered as OLL.

It is accepted that OLL is clinically and histologically similar to OLP [11, 13] but has less characteristic morphology and distribution, whereas lichen planus-like lesions are described as oral lichenoid lesion (OLL), oral lichenoid tissue reaction (OLTR), or lichenoid contact stomatitis [14]. The etiology of OLL is usually identifiable: in case of topographically related lesions to amalgam fillings, intake of particular drugs, history of chronic graft versus host disease (cGvHD), or systemic diseases [4, 15]. OLL is also considered as potentially malignant disorder [4], although it is still unclear whether its malignant potential differs from OLP [16]. So far, various authors [1, 2, 14, 17] have suggested different parameters that might be indicative for OLP and OLL diagnosis (Table 1). Given that the approach to treatment planning and prognosis of both OLP and OLL usually differs, the suggested parameters define criteria that distinguish these lesions.

As opposed to rather evident clinical diagnosis of OLP, less obvious, imprecisely or incompletely defined histopathological findings often complicate the confirmation of final diagnosis. Therefore, the aim of this preliminary study was to determine the correlation of histopathological and clinical diagnosis of OLP and OLL and to clarify which histopathologic features distinguish best these two diagnoses and could improve differential histopathological diagnosis.

## 2. Materials and Methods

The retrospective study group comprised 106 patients who were referred to the Department of Oral Medicine, School of Dental Medicine, University of Zagreb, in the period between January 1, 2012, and December 31, 2012. Diagnosis was based on clinical examination and medical and dental history, intake of drugs, and duration of the lesions. Clinical diagnoses were established by experienced (MMS) and trained clinicians (BLB, IB), specialist in oral medicine who used the same and consistent criteria in scoring levels of OLP and OLL. Histopathologic diagnosis was established at a single pathology service that is under supervision of an experienced pathologist (SS). In all patients with OLP and OLL oral mucosa swabs for yeast culture on Sabouraud Agar plates (Sabouraud Dextrose Agar (Becton Dickinson and Co., Cockeysville, USA) were taken, as a routine procedure in all patients with white oral lesions. Those who have had positive finding of yeast superinfection were excluded from the study. Only patients who underwent biopsy were included in the study. The clinical diagnosis of OLP was established in 92 patients (70 female; 22 male; ratio f/m = 3.18 : 1), while in 14 patients OLL was diagnosed (6 female; 8 male, ratio f/m = 0.75 : 1). The mean age of OLP and OLL patients was 56.1 and 64.9 years, respectively.

OLP was diagnosed according to criteria described by Kramer et al. [10]: presence of white papules and/or striae usually with bilateral involvement and histopathological signs of liquefaction degeneration in the basal cell layer (degenerative changes to the basal cells) along with the presence of a well-defined band-like zone of inflammatory infiltrate confined to the superficial part of the connective tissue (this infiltrate being composed almost exclusively of lymphocytes and characterized by absence of epithelial dysplasia). Clinical diagnosis of OLL was established due to the presence of hyperkeratotic lesions adjacent to amalgam fillings with asymmetric and mainly unilateral distribution and a patient medical history related to drugs, which provoke lichenoid changes in oral mucosa [16].

Data were analyzed by using Chi-squared test (*χ* ^2^) and differences at *P* < 0.05 were considered to be significant.

## 3. Results

**Figure 1:**
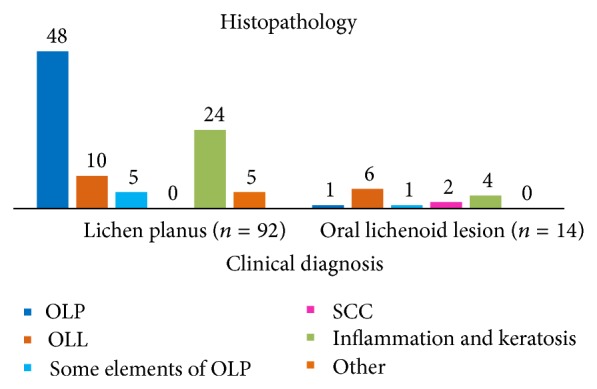
The distribution of clinical and histopathologic diagnoses among patients; OLP: oral lichen planus; OLL: oral lichenoid lesions; OSCC: oral squamous cell carcinoma.

The distribution of clinical and histopathological diagnoses is shown on Figure 1. In 52.2% (48/92) of patients, clinical diagnosis of OLP was histopathologically confirmed, while in 5.4% (5/92) of cases there was a partial confirmation and only some criteria were fulfilled. In 10.9% (10/92) of OLP patients, both clinical and histopathologic diagnosis were concordant, while in 26.1% (24/92) of patients histopathologic diagnosis was nonspecific, being described as inflammation and keratosis. Clinical and histopathological diagnoses coincide in 42.9% (6/14) of OLL patients. According to the histopathologic findings, in clinically diagnosed OLL, in one case the diagnosis of OLP was established, one have had some histopathological elements of OLP, and 4 (28.6%) cases had inflammation and keratosis, while in 2 (14.3%) cases squamous cell carcinoma (OSCC) was diagnosed.

**Figure 2:**
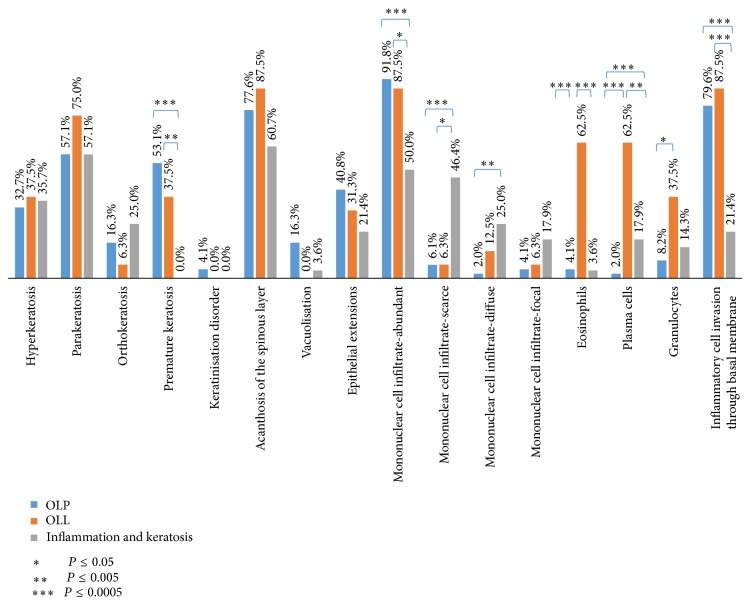
The distribution of histopathologic criteria between the diagnosis oral lichen planus (OLP) and oral lichenoid lesion (OLL).

The distribution of histopathologic features between OLP and OLL with statistically significant differences between the parameters is indicated in Figure 2. Results showed significantly more eosinophils (*P* < 0.0005), plasma cells (*P* < 0.0005), and granulocytes (*P* < 0.05) in OLL than OLP. Other observed parameters such as premature keratosis and inflammatory cell invasion through basal membrane were more frequently found in OLP and OLL in comparison with nonspecific clinical findings of inflammation and keratosis; the difference was significant (*P* < 0.005 and *P* < 0.0005, resp.). Abundant and diffuse mononuclear infiltration was more frequently found in cases of inflammation and keratosis than in OLP or OLL with statistically significant differences (*P* < 0.05, *P* < 0.005, or *P* < 0.0005), but between OLP and OLL diagnosis statistically significant difference in these parameters was not found.

## 4. Discussion

To establish and confirm OLP and OLL diagnosis by using methods such as clinical examination and histopathological analysis, which are available in everyday clinical practice and among wider population of patients, sometimes represents a diagnostic challenge. Earlier reports have shown that while clinical diagnosis depends on a clinician interpretation [11, 14], histopathological diagnosis is strictly dependent on a pathologist interpretation as well [2, 12], but also the choice of biopsy area [18], clinical severity of the disease, activity or remission of the disease, and the clinical type of OLP (reticular lesions are considered easier for histopathological confirmation) [2, 19]. Pathologists' lack of information on clinical features and distribution of lesions could also influence their judgment [11, 12, 20]. Having in mind these parameters, which could affect the final histopathologic interpretation, the results of our study could partially be explained by possible interobserver bias as the patients were examined by different clinicians, and histopathological diagnosis was done by different pathologists. This should be taken into account in the future prospective studies. Therefore, due to many variables affecting diagnosis, histopathological finding is insufficiently reproducible [2, 12].

The results of this study show that clinical diagnoses of OLP and OLL have been confirmed histopathologically in 52.2% patients with OLP and 42.9% patients with OLL. These results are similar to those shown by van der Meij and van der Waal [11] according to whom 42% of OLP clinically diagnosed cases were not confirmed by histopathology. Interestingly, authors also showed that a clinician's consensus in patients with clear histopathological confirmation of OLP was achieved in only 50% of cases. Thornhill et al. [1] showed an overall correlation of clinical and histopathological diagnoses of OLP and OLL based on the findings of five different pathologists. Difficulties reported in distinguishing these lesions in histological features were related to amalgam fillings in only 36% of cases. These results are similar to those of Al-ani [20] who found clinical and pathological correlation of OLP in only 38.5%. Rad et al. [21] found significantly higher clinical and pathological correlation of OLP (93.9%) in cases in which WHO modified criteria were applied. The results of Rad et al. [21] are promising but should be validated in a larger group of patients, applying strictly validated and reproducible criteria [11].

According to the literature review, there is a lack of studies, which applied WHO modified criteria in determining correlation of clinical and histopathological diagnosis of OLP. It would be reasonable to expect that the overall prevalence of OLP in the observed population would be lower and the prevalence of OLL higher if WHO modified criteria for establishing diagnosis were applied. This could possibly affect an expected malignant transformation rate in both OLP and OLL showing that OLL is more prone to malignant transformation than OLP as already documented by some authors [8, 22] and also shown in our results.

Van der Meij et al. [23, 24] have found in prolonged follow-up that patients with OLL have an increased risk for malignant transformation compared to OLP and that this is more likely to occur in erosive lesions. The malignant transformation could be attributable to the extrinsic factors [24], other than alcohol and tobacco, use of particular antihypertensive [25] and antiretroviral drugs [26], and infection with strains of* Candida albicans* [27], which all can enhance malignant transformation in oral lesions. In our two patients with oral SCC we did not found any of the listed risk factors. This finding emphasises the importance of regular follow-up of patients with oral lesions and eventually biopsies to confirm or refute clinical diagnosis of OLL or OLP.

Gannot et al. [28] have shown that tissue changes towards malignancy had a distinct lymphocyte profile: the number of CD4, CD8, and B cells is significantly higher in inflammatory infiltrate in moderate and severe dysplasia and SCC, compared to mild dysplasia and hyperkeratosis. In addition, inflammatory cell infiltration has been considered in cancer progression. Particularly, CD8 and NK cell are increased in oral SCC since they have cytotoxic immune response against neoplastic cells [29].

Several authors so far have suggested histopathologic criteria, which should be distinctive in achieving both OLP and OLL diagnoses (Table 1). In their studies, they found differences in some parameters between OLP and OLL, but in order to accept these findings as a standard protocol, a larger number of patients should be examined as already suggested by some authors. All authors agree that findings of plasma cells, eosinophils, and neutrophils in inflammatory infiltrate can be distinctive for OLL compared to OLP, which is in consensus with our results (Figure 2). Therefore, we consider that this type of cells should be a certain diagnostic feature in histopathologic differentiation between OLP and OLL given that these cells are relevant in nonimmediate allergic reactions, particularly those induced by drugs [30, 31], where OLL is classified.

Unlike other findings (Table 1), we did not observed statistically significant differences in other histopathological criteria between OLP and OLL. These results therefore may enhance a closer collaboration with pathologists in order to get a more precise description of histopathological features, particularly cellular composition of mononuclear infiltrate. This consequently should improve the list of parameters, which pathologists may consider relevant for either diagnosis, therefore avoiding misinterpretation due to different criteria, which usually affect the results.

## 5. Conclusion

OLP and OLL are clinically similar but diagnostically insufficiently distinguished lesions. Their prognosis and treatment may vary. To achieve accurate histopathologic diagnosis it is mandatory to define the type of cells in mononuclear infiltrate, which can be interpreted in accordance with clinical findings and a patient history. Currently accepted diagnostic criteria in diagnosing OLP and OLL should be modified and modifications should be validated in a larger number of patients. The results of this study, which is in accordance with similar reports from the literature, have shown that histopathological findings coincide with clinical diagnosis in approximately only 50% of cases and as such cannot always be exclusive in the final interpretation. Based on the results of this study, prominent diagnostic histopathological features in distinguishing between OLP and OLL are the type of cells in the mononuclear cell infiltrate, that is, eosinophils, plasma cells, and granulocytes.

## Figures and Tables

**Table 1:** Histopathological criteria distinctive of OLP and OLL.

Diagnosis	Epithelium	Subepithelium	Authors
OLP	Bilateral presentation of lesions Hyperkeratosis Reduced epithelial thickness Liquefactive degeneration of the basal cell layer	Well-defined subepithelial band of chronic inflammatory infiltrate composed predominantly of lymphocytes Absence of eosinophils and neutrophils	Juneja et al., 2006 [14]
		
		Band shaped inflammatory infiltrate in some or all areas	Thornhill et al., 2006 [1]
		
	Normal stratification and maturation Basal cell liquefaction is always present Atypia absent	Dense band of inflammatory components, chiefly lymphocytes, in the juxtaepithelium Dyskeratotic epithelial cells Civatte bodies are usually found in subepithelial area, at the junction of the lamina propria and epithelial layer	Ismail et al. 2007 [22]
		
	Compact hyperorthokeratosis, seldom a moderate degree of parakeratosis Thickened stratum granulosum at acrosyringia and acrotrichia, irregular saw-tooth-like epidermal layers	Mostly a superficial dermal inflammatory infiltrate; seldom eosinophils acanthosis, necrotic keratinocytes in the lower epidermal layers	Ziemer, 2014 [17]

OLL	Unilateral presentation of lesions	Poorly differentiated lower border of the subepithelial inflammatory zone Presence of a substantial number of plasma cells in the lymphocytic infiltrate Perivascular infiltrate Increased number of colloid bodies Presence of acute inflammatory cells, such as eosinophils and neutrophils	Juneja et al., 2006 [14]
		
	Focal parakeratosis, cytoid bodies in the cornified layer Thickened stratum granulosum possible, however, with focal interruption of the granular layer, cytoid bodies in the granular layer, necrotic keratinocytes scattered in all epidermal layers	More often a deep dermal infiltrate, especially in nonphotodistributed lichenoid drug eruption; admixture of eosinophils and plasma cells possible (presence of plasma cells is a regular finding in biopsies from mucous membranes independently of the origin of dermatosis)	Ziemer, 2014 [17]

OLL related with amalgam filling		Inflammatory infiltrate located deep to superficial infiltrate in some or all areas Focal perivascular infiltrate Plasma cells, eosinophils, and neutrophils in the connective tissue	Thornhill et al., 2006 [1]
		
	Normal stratification basal cell liquefaction may or may not be present Atypia absent	Lymphoid follicle formations, with mixed inflammatory cells consisting of plasma cells and neutrophils	Ismail et al. 2007 [22]
		
	Basal cell liquefaction may not be present	Predominant formation of lymphoid follicles chiefly consisting of plasma cells and neutrophils Dense inflammatory cells in the stroma	Hiremath et al., 2011 [2]

OLL related with drugs	Extensive degeneration in the lower prickle cell layer, prompting spongiotic vesicle formation Basal cell liquefaction is usually present Atypia absent Apoptotic and colloid body formation are evident	Infiltrate is not band-like but extends to the deeper stroma inflammatory cells predominated by plasma cells and eosinophils Perivascular cuffing of inflammatory cells is evident	Ismail et al. 2007 [22]
		
		Infiltrate is often not band-like but extends to the deeper stroma, with plasma cells and eosinophils which predominate the inflammatory component	Hiremath et al., 2011 [2]

OLP: oral lichen planus; OLL: oral lichenoid lesions.
